# Functional capacity and quality of life in the postural tachycardia syndrome: A retrospective cross-sectional study

**DOI:** 10.1016/j.amsu.2020.06.013

**Published:** 2020-06-12

**Authors:** Erika Hutt, Ramya Vajapey, Erik H. Van Iterson, Fredrick Jaeger, Leslie Cho, Haitham M. Ahmed, Kenneth A. Mayuga

**Affiliations:** Heart and Vascular Institute, Cleveland Clinic, Cleveland, OH, USA

**Keywords:** Postural tachycardia syndrome, Functional capacity, Quality of life, Age, Gender

## Abstract

**Background:**

Postural tachycardia syndrome (POTS) is a complex syndrome of orthostatic intolerance that significantly affects quality of life. The relationship between functional capacity, quality of life, and age remains poorly understood in this patient population. The purpose of this cross-sectional study was to assess the clinical and exercise characteristics of patients with POTS who underwent exercise stress testing as part of cardiac rehabilitation, and to evaluate the relationships between functional capacity with age and sex, as well as the relationship between functional capacity and quality of life.

**Methods:**

We included 255 consecutive adult patients with the diagnosis of POTS, by tilt table testing, who underwent exercise stress testing between 2012 and 2017. Clinical and stress test data were obtained from electronic medical records.

**Results:**

Of the 255 patients, 232 (91%) were women, with median age 33.5 years. Prevalence of traditional CAD risk factors was low (2% diabetes, 13% hypertension, 7% hyperlipidemia, and 26% smoking history). Mean resting BP was 114 ± 13 mmHg systolic, resting HR was 76 ± 13 bpm, METs achieved 9.2 ± 2.2, and 1 min HR recovery 32 ± 17 bpm. 113 patients (44%) had abnormal functional capacity for age and sex. When analyzed by age groups, younger POTS patients had increasingly lower than expected functional capacity (compared to predicted normals) than did older patients (ANOVA *P* = 0.0017). The SF-36 physical component of patients with abnormal functional capacity was significantly lower than those with normal functional capacity. (p = 0.006).

**Conclusions:**

In this large cohort, patients with POTS were predominantly female (91%) and relatively young. The novel findings are that younger patients with POTS were more likely to have lower-than-average functional capacity for their age and sex compared to older patients, and that abnormal functional capacity was associated with lower quality of life by SF-36 physical component.

## Highlights

• Postural tachycardia syndrome (POTS) is a complex and poorly syndrome of orthostatic intolerance that significantly affects quality of life.
• In this study, younger patients with POTS were more likely to have lower-than-average functional capacity for their age and sex compared to older patients.
• In this study, abnormal functional capacity was associated with lower quality of life by SF-36 physical component.

## 1 Introduction

Postural tachycardia syndrome (POTS) is a complex, poorly understood syndrome characterized by chronic orthostatic intolerance that significantly affects quality of life. It is associated with symptoms of lightheadedness, fatigue, blurred vision and exaggerated increase in heart rate while standing, in the absence of orthostatic hypotension [1,2]. POTS is defined by the presence of chronic orthostatic intolerance accompanied by an increase in heart rate (HR) of ≥30 bpm or by a HR of ≥120 bpm after standing, in the absence of orthostatic hypotension [[2], [3], [4], [5]].

The prevalence of this heterogeneous clinical syndrome is unknown but estimated to be at least 170/100,000, affecting between 0.1 and 1% of the United States population [[6], [7], [8]]. It has a strong female predominance with a female to male ratio of 5:1 and primarily affects women of childbearing age [3,9].

The pathophysiology remains unknown but many theories have been described including hypovolemia, autonomic dysfunction, hyperadrenergic state, adrenergic receptor deficiency, mast cell activation, renin-aldosterone paradox and physical deconditioning [10]. This may explain why various treatment options have been used for the management of POTS, but with suboptimal results. Studies have reported improvement in symptoms with saline infusion [11], beta blockers [12], erythropoietin [13] and progressive exercise training [14]. The latter has shown to improve exercise capacity, left ventricular diastolic function and quality of life, with variable improvement in standing hemodynamics [14,15].

A better understanding of exercise physiology in this population is required to move forward in the management of this complex syndrome. The purpose of this study was to assess the clinical and exercise characteristics of patients with POTS who underwent exercise stress testing as part of the evaluation for cardiac rehabilitation, and to evaluate the relationship of functional capacity with age and sex, as well as the relationship between functional capacity and quality of life.

## 2 Methods

Adult patients with the diagnosis of POTS, with a consistent clinical scenario combined with results from Tilt Table Testing, who underwent exercise stress testing at our institution as part of the evaluation for cardiac rehabilitation were included in this retrospective cross-sectional chart review study. This study was reviewed and approved by the Institutional Review Board of Cleveland Clinic, with waiver of informed consent (reference number 17-1067). This study was registered with Research Registry (unique identifying number of research registry 5523). Between 2012 and 2017, 255 consecutive adult patients with the diagnosis of POTS, by Tilt Table Testing, who underwent exercise stress testing were included. Of note, as has been the practice at our institution, both ‘Early POTS’ (a diagnostic heart rate increase occurring within 10 min of upright tilt) and ‘Late POTS’ (occurring after 10 min of upright tilt) were included in the diagnosis of POTS in the setting of a consistent clinical scenario [15]. Clinical and stress test data were obtained from digital records. Exercise stress tests were performed following Bruce or Cornell protocols. Normal functional capacity was defined as average or above average METs achieved during exercise stress test (see Supplementary Table S1 for definition of average METs by age and sex). The patients completed SF-36 questionnaires as part of the routine history taking before undergoing exercise stress testing.

### 2.1 Statistical analysis

Continuous variables are expressed as mean values ± standard deviation (SDs) or median and quartiles, and categorical variables are presented as absolute numbers and percentages (%). Categorical variables were compared using chi-square and continuous variables were analyzed using independent *t*-test or ANOVA for >2 variables. SF-36 questionnaire scores of patients with normal vs abnormal functional capacity were compared using independent sample *t*-test [16].

Functional capacity was analyzed as a categorical variable (classified in two groups: below average functional capacity and above average functional capacity) as well as a continuous variable based on METs. When comparing functional capacity as a nominal variable with two categorical variables (i.e age, blood pressure, heart rate, etc) an independent *t*-test was used. When comparing functional capacity based on METs as a continuous variable for more than 2 groups, ANOVA was used.

## 3 Results

**Fig. 1:**
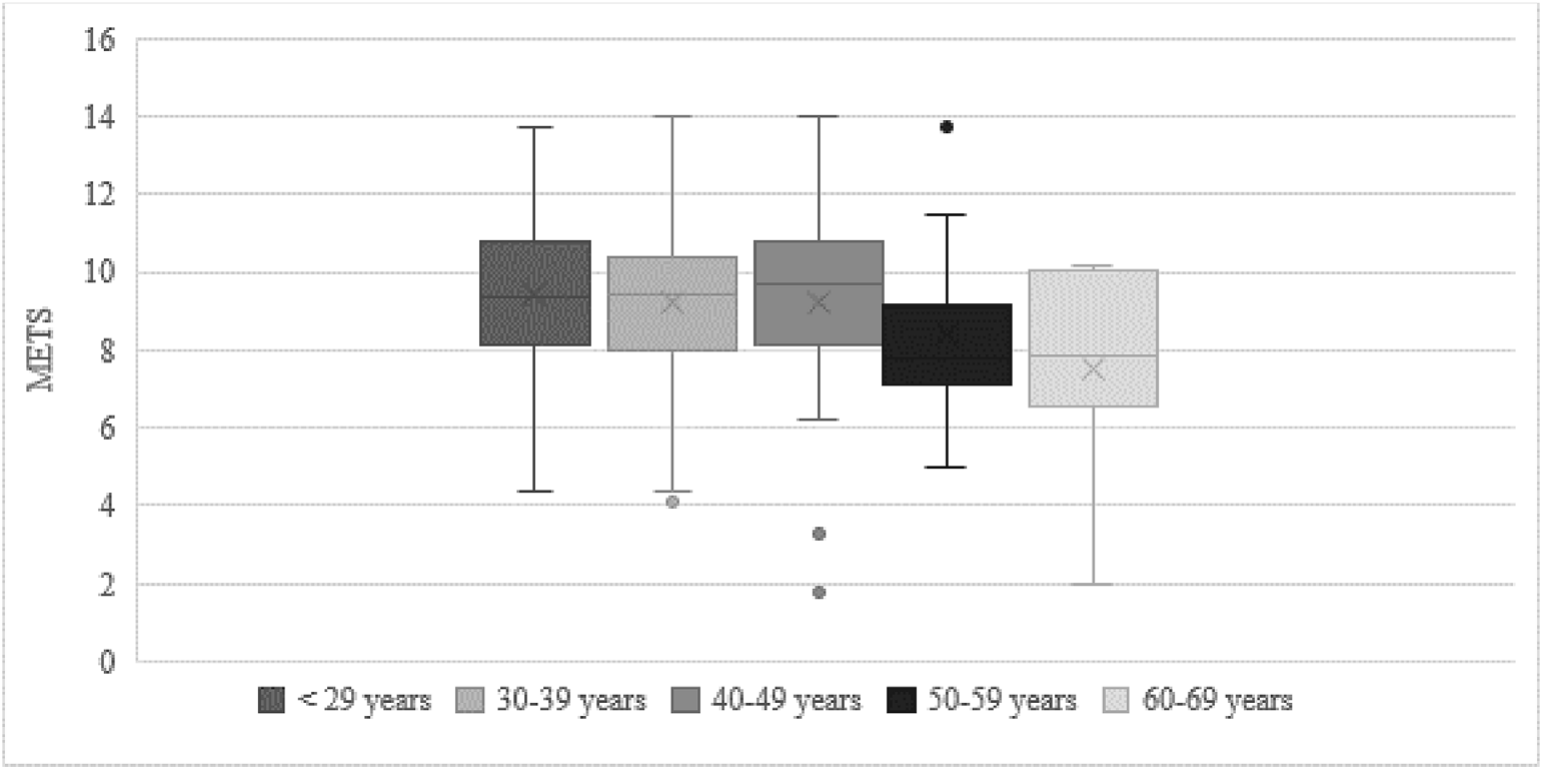
METs achieved by age group.

A total of 255 patients with POTS who underwent exercise stress testing between 2012 and 2017 were included. Patients’ mean age was 33.5 ± 11.3 years. Of the 255 patients, 232 (91%) were women. Baseline characteristics are shown in Table 1. Prevalence of traditional CAD risk factors was low (2% diabetes, 13% hypertension, 7% hyperlipidemia, and 26% smoking history). The onset of POTS during tilt table testing (achieving a diagnostic heart rate increase) occurred at a mean of 17.5 ± 10.1 min of upright tilt. Stress test data is shown in Table 2. Of the 255 patients, 113 patients (44%) had abnormal functional capacity for age and sex. METs achieved by age groups are shown in Fig. 1.

**Table 1:** Baseline clinical characteristics of study population.

Characteristic	POTS population (N = 255)
Age	33.5 ± 11.3
Female gender	232 (91%)
BMI	25.9 ± 10.6
Diabetes Mellitus	5 (2%)
Hypertension	33 (13%)
Hyperlipidemia	18 (7%)
Family history of CAD	50 (20%)
Smoking history	66 (26%)
Mean SF-36 (physical component)	30.5 ± 9
Mean SF-36 (mental component)	44.6 ± 10

BMI: body mass index; CAD: coronary artery disease.

**Table 2:** Stress test characteristics of study population.

Stress test characteristic	POTS population (N = 255)	P value (independent sample *t*-test)
Resting BP	114.1 ± 13.3	
Peak BP	147.7 ± 23.3	
Δ in BP	33.6	p < 0.001
Resting HR	75.5 ± 13.4	
Peak HR	170.6 ± 21.4	
Δ in HR	95.1	p < 0.001
% Maximum HR	91.6 ± 11.0%	
HRR	31.9 ± 16.8	
CRI	0.86 ± 0.18	
Exercise Time	540.1 ± 140.2	
METS	9.2 ± 2.2	

BP: blood pressure, HR: heart rate, HHR: heart rate recovery, CRI: chronotropic response index.

**Fig. 2:**
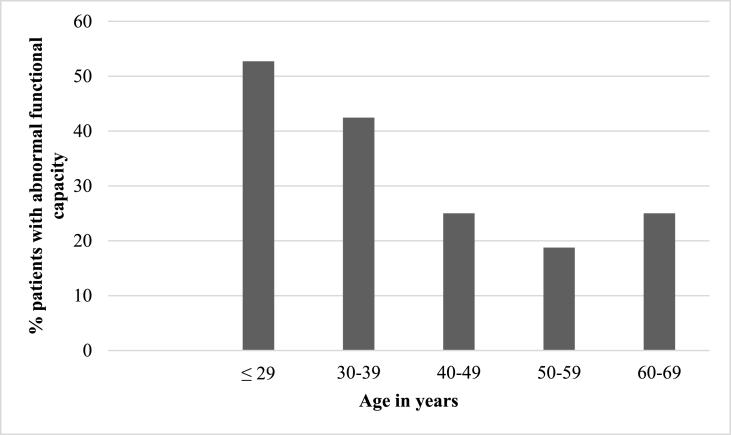
Prevalence of abnormal functional capacity by age group. *See Supplemental material for thresholds used to define abnormal functional capacity by age groups.

When analyzed by functional capacity, patients with below average achieved METs (n = 113) were younger (30.9 vs. 35.6 years, p < 0.001), had lower peak systolic blood pressure (143 vs. 151 mmHg, p = 0.006) and heart rate (164 vs. 176 beats per minute, p < 0.001), lower chronotropic response index (0.78 vs. 0.93, p < 0.001), and shorter exercise time (447 vs. 615 s, p < 0.001). Table 3 reports the clinical and stress test characteristics of patients based on functional capacity. When analyzed by age groups, younger POTS patients had increasingly lower than expected functional capacity by METs (compared to predicted normal functional capacity, reference available in supplement material) than did older patients (ANOVA *P* = 0.0017, Fig. 2).

**Table 3:** Clinical and stress test characteristics of study population based on functional capacity.

Characteristic	Below average FC (n = 113)	Average or above average FC (n = 142)	p value
Age	30.9 ± 9.9	35.6 ± 11.9	p < 0.001
Female Gender	99	133	p = 0.1
BMI	25.8 ± 6.3	26.0 ± 13.1	p = 0.8
Diabetes	0	4 (2.8%)	p = 0.2
Hypertension	15 (13.3%)	18 (12.7%)	p = 0.9
Hyperlipidemia	10 (8.9%)	8 (5.6%)	p = 0.5
FH CAD	24 (21.2%)	26 (18.3%)	p = 0.7
Smoking history	31 (25.2%)	35 (24.6%)	p = 0.7
Resting SBP	114 ± 12	114 ± 14	p = 0.9
Peak SBP	143 ± 24	151 ± 22	p = 0.006
Resting HR	77 ± 15	75 ± 12	p = 0.2
Peak HR	164 ± 24	176 ± 18	p < 0.001
HRR	40 ± 0	40 ± 3	p = 0.9
% Max HR	95.4 ± 8.2	86.7 ± 12.1	p < 0.001
CRI	0.78 ± 0.20	0.93 ± 0.14	p < 0.001
Exercise Time	447 ± 115	615 ± 111	p < 0.001
METS	7.5 ± 1.7	10.5 ± 1.50	p < 0.001

*BMI: body mass index, SBP: systolic blood pressure, HR: heart rate, HRR: heart rate recovery, CRI: chronotropic response index.

**Fig. 3:**
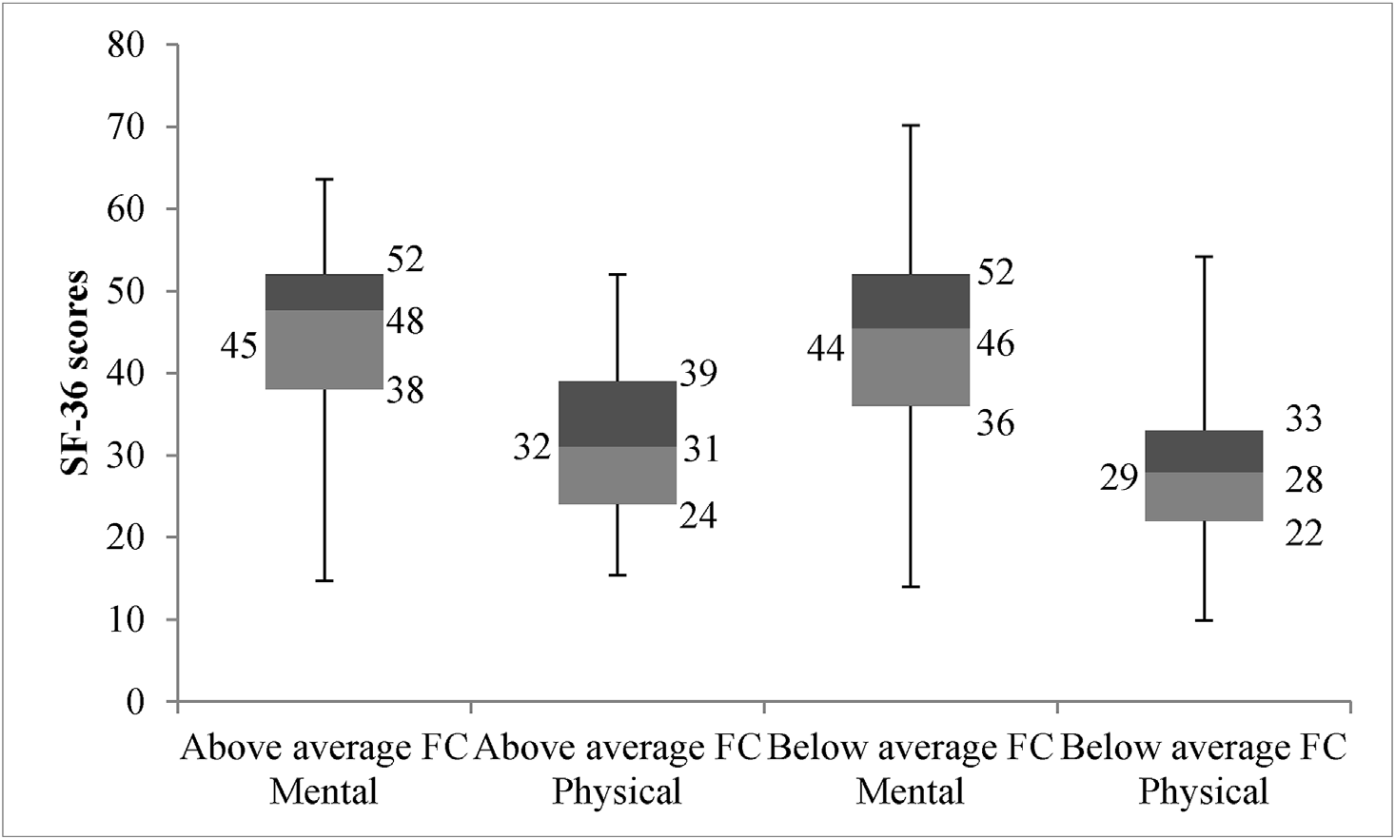
Relationship between SF-36 & Functional Capacity. *Mean values are shown on the left of the box plots and quartiles with median values are shown on the right of the box plots.

The mean SF-36 scores for mental and physical components were 44 ± 11 and 28.6 ± 9.2 respectively in patients with low functional capacity compared to 45.2 ± 10.2 and 32.4 ± 9.1 respectively in those with normal functional capacity. The SF-36 physical component of patients with abnormal functional capacity was significantly lower than of those with normal functional capacity. (28.6 ± 9.2 vs 32.4 ± 9.1, p = 0.006) (Fig. 3). No difference was found in the SF-36 mental component between the 2 groups.

## 4 Discussion

In this study, we sought to investigate the functional capacity of patients with POTS and the relationship to quality of life. The major novel findings are that (1) younger patients with POTS were more likely to have lower-than-average functional capacity for their age and sex compared to older patients, and (2) abnormal functional capacity was associated with lower quality of life by SF-36 physical component.

Obidoa et al. conducted a structured review of longitudinal studies that reported SF-36 scores among healthy individuals between the age of 18 and 65. Their report summarizes the mean SF-36 scores observed in this population and found that mean SF-36 scores for physical component range between 83 and 91 for women and men respectively. In our cohort, the mean SF-36 score for physical component was 30.5. Despite our inability to compare these using statistical tests, we can say that this score is considerably lower than that seen in the healthy general population. Likewise, mean SF-36 scores for mental component ranged between 69 and 76 in the general population per the study by Obidoa et al. In contrast, we observed a mean SF-36 score for mental component of 44 in our cohort. This again reflects an overall poor quality of life of patients with POTS [17].

Exercise training has been shown to be effective in improving quality of life in these patients [18,19]. However, the compliance to exercise training is estimated to be around 40-50% and its long-term benefits remain unknown. Thus, this patient population continues to pose significant challenges to the treating physician, who struggles to offer effective treatment. The fact that younger patients have lower-than-average functional capacity (compared to age- and sex-matched normals) versus older patients may suggest either that the earlier onset of POTS is more debilitating or that the syndrome improves with aging (with respect to functional capacity) [[20], [21]].

Furthermore, the study showed that there is an association between functional capacity and quality of life. Studies evaluating the effectiveness of exercise training in POTS have shown that virtually every patient who completes the exercise program has improvement in quality of life by SF36, but not necessarily in the heart rate response to standing [22]. In this study, despite the fact that exercise effectiveness was not being tested, we found that normal functional capacity is indeed associated with better quality of life in the SF36 physical component, supporting the recommendation of exercise training in POTS to improve functional capacity. However, the mental component of the SF 36 was not associated with functional capacity, a phenomenon which needs further investigation and which highlights the complex nature of POTS.

### 4.1 Limitations

This study is limited by the observational nature of a registry, which makes it prone to selection and referral biases. It is also limited by the fact that the stress test obtained shows the functional capacity at one single point in time, thus little can be said about the long term effect of exercise. Additionally, we did not have information about the time of enrollment of each individual in this exercise program so duration, compliance and stage of training in our exercise program could have been heterogeneous between patients.

Despite this being one of the largest POTS registries reported in literature, it still has a low sample size which did not allow more complex statistical analysis. That being said, the descriptive findings of this study are of importance given that this population faces many challenges, many of which are due to the lack of understanding of this condition.

## 5 Conclusion

In this large clinical cohort, patients with POTS were predominantly female (91%) and relatively young. The novel findings are that younger patients with POTS were more likely to have lower-than-average functional capacity compared to older patients, and that abnormal functional capacity was associated with lower quality of life by SF-36 physical component.

## Ethical approval

This retrospective study was reviewed and approved by the Institutional Review Board of Cleveland Clinic, with waiver of informed consent, approved on 8/11/2017 (Reference number 17-1067).

## Funding sources

Nothing to declare.

## Author contribution

Erika Hutt, MD: study concept, study design, data collection, data analysis, writing/reviewing. Ramya Vajapey, MD: study concept, study design, data collection, data analysis, writing/reviewing. Erik H. Van Iterson, PhD: writing/reviewing. Fredrick Jaeger, DO: writing/reviewing. Leslie Cho, MD: writing/reviewing. Haitham M. Ahmed, MD: study concept, study design, data collection, data analysis, writing/reviewing. Kenneth A. Mayuga, MD, FHRS, FACC: study concept, study design, data collection, data analysis, writing/reviewing.

## Registration of research studies

Name of the registry: Research Registry.

Unique Identifying number or registration ID: researchregistry5523.

Hyperlink to your specific registration (must be publicly accessible and will be checked): https://www.researchregistry.com/browse-the-registry#home/registrationdetails/5e9f108f65596e0017b9a856/

## Guarantor

Kenneth A. Mayuga, MD.

## Provenance and peer review

Not commissioned, externally peer reviewed.

## Consent

This retrospective study was reviewed and approved by the Institutional Review Board of Cleveland Clinic, with waiver of informed consent, approved on 8/11/2017 (Reference number 17-1067).

## Declaration of competing interest

Nothing to declare.
