# Supplementary material for: Functional capacity and quality of life in the postural tachycardia syndrome: A retrospective cross-sectional study
Source: Ann Med Surg (Lond). 2020 Jun 12;56:72–6. doi: 10.1016/j.amsu.2020.06.013 (PMC7322180; doi:10.1016/j.amsu.2020.06.013)
Supplement: Multimedia component 2 [file mmc2.docx]

**Supplementary material**

Table S1. Cutoffs for average functional capacity based on gender and age used at our institution.

| Women age range | Average Functional Capacity (METs) | Men age range | Average Functional Capacity (METs) |
| --- | --- | --- | --- |
| ≤29 | 10-13 | ≤29 | 11-14 |
| 30-39 | 9-11 | 30-39 | 10-12.5 |
| 40-49 | 8-10 | 40-49 | 8.5-11.5 |
| 50-59 | 7-9 | 50-59 | 8-11 |
| 60-69 | 6-8 | 60-69 | 7-9.5 |
| 70-79 | 4.5-6.5 | 70-79 | 5.5-8 |
| ≥80 | 4-4.5 | ≥80 | 4.5-6.5 |
